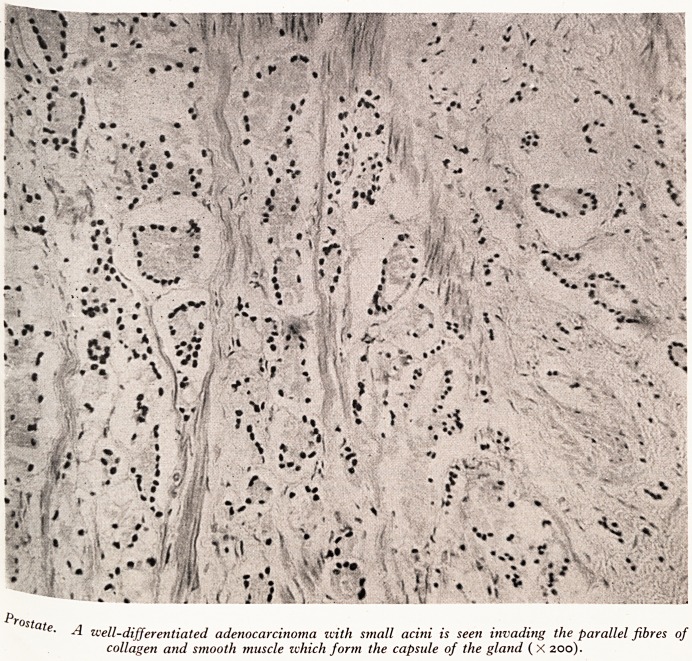# Primary Myelosclerosis with Leuco-Erythroblastic Anaemia

**Published:** 1956-01

**Authors:** O. C. Lloyd


### PRIMARY MYELOSCLEROSIS WITH LEUCO-ERYTHROBLASTIC ANAEMIA

*A Clinical Pathological Conference of the University of Bristol Medical School on 16th November, 1954*

CHAIRMAN: DR. O. C. LLOYD

*Dr. O. C. Lloyd:* The case tonight is that of a man of 74, a retired solicitor's clerk. His general practitioner was Dr. P. N. Heron, who is unable to be present tonight but he has written me a letter, in which he says that although the man was a patient of his for almost ten years, he saw him hardly at all. He did, however, see him in 1946 or 1947 and at that time he refused to have anything done and would not go to out-patients or have any private opinion. His attitude was " I don't care if it kills me as long as there is no pain". Then early in 1954 he got acute abdominal pain for which Dr. Heron sent him to the Bristol Royal Infirmary, as a possible case of acute intestinal obstruction. " I don't suppose I was so much worried," Dr. Heron wrote, " about the pain as the fact that this was a good opportunity to get this stubborn man into hospital for investigation. However he had me beat and walked out the next day. I saw him a few times in the last two or three months of his life. Always he absolutely refused to go into hospital. It was only during this time that he complained of cough which annoyed him and an obvious increasing weakness but no pain. In the end he was sent to hospital almost in a dying state, because he could no longer get out of bed and his daughter and relatives more or less forced him to go."

He was first admitted with acute abdominal pain on 27th January, 1954, and was under the care of Mr. Cooke, who cannot remember anything about him as he was only in hospital for one day. He was readmitted on 15th August, 1954, and this time he was under Dr. Cates, who will give the main points of the story.

*Dr. J. E. Cates:* This man was admitted as an emergency because he was cachectic and obviously about to die. He was very wasted and profoundly anaemic; he had difficulty in passing water and was complaining of a lump in his abdomen. The history obtained at the time was that ten years previously he had himself noticed the lump. Four years before he came in, he began to feel easily tired and began getting thinner and pale. You have already heard how eight months previously he had been admitted with sudden left-sided abdominal pain, so severe and sharp, that he was thought to have an abdominal emergency, possibly intestinal obstruction. He was at once found to have a very large spleen. A blood count showed that he had a lot of primitive cells in his peripheral blood and his haemoglobin was only 66 per cent. He was thought to have myeloid leukaemia. He went home before full investigations could be made and slowly got worse. He became even paler and thinner and three weeks before readmission he had pain in the legs, collapsed and took to his bed. For the last two weeks before admission he was in bed all the time. On admission he was very thin and frail, and so weak that he could hardly move his arms and had to be fed. He was ghastly pale with a cough and had difficulty in breathing. The main physical signs were that he had a very large spleen which came right down into the left iliac fossa and a large liver, about five fingers' breadth below the right costal margin. There were no signs of leukaemia other than the large spleen and liver; there were no enlarged lymph nodes; his gums were not swollen or infected and there was no sore throat. At the time the diagnosis still seemed to be myeloid leukaemia and relevant investigations were put in hand. The haemoglobin was 22 per cent.; the red cells were not counted but the appearance of the blood film was that of hypochromic anaemia. There were many nucleated cells. The total white cells were 40,000 per cu. mm. of which 30,000 were polymorphs. There were a few myeloblasts and normoblasts and immature cells of all series were seen in the blood film, which meant that he had a leuco-erythroblastic anaemia. The blood films made in January 1954 were re-examined and were found to support this diagnosis, since they also showed primitive red and white cells. So we had another look at the patient. Here was a man who was very anaemic with probable leuco-erythroblastic anaemia: what could be the cause of it? The pathologists tell us that primitive red and white cells appear in the peripheral blood when the bone marrow is stimulated and invaded by disease, such as secondary carcinoma, Hodgkin's disease or rare bone diseases; also myelosclerosis—a disease in which there is obliteration of bone marrow by fibrous tissue. What could the cause be in this case? He had had some difficulty in passing water and his prostate was found to be hard and craggy and enlarged: we therefore thought it most likely that the disease of the bone marrow was infiltration by secondaries from a primary cancer of the prostate. We did not X-ray his bones as he was too ill. A bone marrow biopsy was done but there were no cancer cells present. On this rather thin presumptive diagnosis we put him on stilboestrol. He refused to have any blood transfusions or " mucking around ". He became more oedematous, he had a bedsore, and became weaker and drowsier and eventually died thirteen days after admission. Before the post-mortem was done the feeling was that this was a man with leuco-erythroblastic anaemia, due probably to carcinoma of the prostate. But he had had a big spleen for a long time and an attack of perisplenitis, presumably, eight months before. One would not expect this with fairly recent onset of infiltration of bone marrow; and these two things, clinically, we could not put together.

*Dr. R. Moyle* showed some X-rays: In the chest X-ray no deposits were seen in the lungs, mediastinum or ribs. An X-ray of the right humerus showed that a portion was decidedly sclerotic and in the femur the trabeculae were very much coarsened and roughened, with a translucent area.

Dr. Lloyd asked Dr. Johnson to tell the meeting about the haematological investigations.

*Dr. D. H. Johnson:* I first saw a blood film in January 1954 when the total nucleated cell count was 32,000 per cu. mm., mainly polymorphs with myelocytes and more primitive forms present. Although this initially suggested leukaemia a difficulty arose because nucleated red cells were also present in moderate numbers. Myeloid leukaemia, however, especially in its terminal stages does occasionally produce a leuco-erythroblastic picture. I went to see the patient immediately only to find he had discharged himself. He came in again in August and I saw another blood film. The anaemia was more severe, the nucleated white cell count was about the same and the number of primitive white cells and nucleated red cells had increased. With the fuller history now available this appeared to be a true leuco-erythroblastosis: leukaemia no longer suggested itself. From the sternum I obtained a very poorly cellular aspirate with no abnormal cells. As this might not have been a representative sample, I repeated the marrow aspiration at the iliac crest and obtained a similar sample. This tended to confirm the suspicion of generalized fibrosis of the marrow with enlargement of liver and spleen through displacement of haemopoietic activity to these organs. If haemopoiesis is going on outside the marrow, primitive red and white cell precursors can no longer be held back and they appear in large numbers in the peripheral blood. The commonest causes of displacement of the marrow are carcinomatosis and myelosclerosis. Failure to find carcinoma cells did not rule out metastases from carcinoma of the prostate since these may produce an extreme degree of fibrosis with very scanty cells.

*Mr. R. V. Cooke:* Who described the prostate as craggy?

*Dr. Cates:* I did. It was hard and irregular and highly suspicious of carcinoma of the prostate: it was a tentative clinical diagnosis.

*Question:* Was any laboratory test done to support this diagnosis of the carcinoma?

*Dr. Cates:* Yes—a serum acid phosphatase was 5 units.

*Question:* What do you consider to be a diagnostic level?

*Dr. G. K. McGowan:* A serum acid phosphatase over 10 units is almost certainly diagnostic of prostatic carcinoma.

*Mr. Cooke:* How often do you find that in carcinoma of the prostate?

*Dr. McGowan:* I do not know: it is my impression that not more than one-third of the true carcinomas of the prostate have a diagnostically raised acid phosphatase.

*Dr. Lloyd:* Would it not be true to say that when the carcinoma has already metastasized the proportion of the cases then which show a raised acid phosphatase is very much higher?

*Dr. McGowan:* Yes, but this does not necessarily follow, of course, in each individual case.

*Question:* How common is leuco-erythroblastic anaemia?

*Dr. Johnson:* It is slightly more common than leukaemia. The presence of a few myelocytes and an occasional red cell in the blood film from a patient who is known to have, say, carcinoma of the bronchus, is strong evidence that there are in fact secondary deposits in the bone and that any operation is likely to prove only palliative. This is not an infrequent finding in proved cases of carcinoma. It is important to be on the look out for them because of their prognostic significance.

*Dr. Lloyd* (presenting the post-mortem findings):

This man was very much wasted with bedsores over the sacrum, heel and right hip. The pleural cavities were obliterated by fibrous adhesions and there was also slight interlobular fibrosis in the lungs. The abdomen showed extensive fibrous adhesions between the spleen, the omentum and the abdominal wall. They probably represented the healing lesions of the perisplenitis which had caused him the acute abdominal pain.

The heart was enlarged and weighed 385 grammes, the enlargement being mainly due to well-marked dilatation of all the chambers; this dilatation was so great that I was unable to observe any very obvious thickening of the heart muscle itself. There was some white adherent thrombus in the right auricle. I could not see any myocardial pallor attributable to fatty infiltration, but I have no doubt that this heart was dilated and acted poorly because of the long standing severe anaemia.

The lungs showed chronic vesicular emphysema, and severe pulmonary oedema, which was probably terminal and due to heart failure. No metastases of any sort were found in the lumgs.

The spleen was greatly enlarged, weighing 1,640 grammes, and there was well marked perisplenitic fibrosis. It was tough and the cut surface showed a blotchy appearance from areas of cellular hyperplasia congested with blood.

The bones showed well-marked hyperostosis of the medullary trabeculae; even in the shaft of the femur, where the marrow is usually soft, it was ossified. Lilac-coloured marrow extended the full length of the shaft. On microscopy there was fibrosis of the marrow with formation of osteophytes on the inner aspect of the cortex and on the medullary trabeculae (Plates I and II).

The liver weighed 2,480 grammes, which is nearly double the normal figure, and histologically showed obvious foci of myelopoiesis in its sinusoids. The red and white cell precursors tended to be aggregated in separate groups.

The prostate was enlarged and firm, but did not have a nodular pattern. There was no naked-eye infiltration of the capsule, but a section (Plate III) showed that it was in fact invaded by a well-differentiated adenocarcinoma. In spite of this no metastases were found in any organ of the body.

In this case, therefore, there were two unrelated conditions. The man probably had leuco-erythroblastic anaemia due to sclerosis of the bone marrow for a long time. The fact that he had a carcinoma of the prostate had made the diagnosis difficult. The disease was a primary myelosclerosis with leuco-erythroblastic anaemia and enlargement of the spleen.

Myelosclerosis might be due to secondary carcinoma or reticulosarcoma: primary sclerosis is much more rare and this is the first case we have had in the post-mortem room.

*Dr. Cates:* The puzzle about leuco-erythroblastic anaemia is the anaemia; because even with complete replacement of bone marrow there should still be enough haemopoietic tissue, since the liver is enlarged down to the umbilicus and the spleen down to the left iliac fossa. It is said that the spleen becomes the site of red cell formation because the bone marrow is replaced by fibrous tissue, and that therefore it should never be removed in cases of myelosclerosis. There is some evidence that this theory is wrong.

*Dr. Lloyd:* Some people have questioned it and claim that the patient is better when the spleen is removed. Perhaps Dr. Cosh will give us his views on the matter.

*Dr. J. A. Cosh:* I recently saw a case demonstrated at the Johns Hopkins Hospital in whom splenectomy had been performed in the presence of myelosclerosis. They called it " agnogenic myeloid metaplasia " which I discovered to be a phrase meaning myelosclerosis with leuco-erythroblastic anaemia of unknown cause. The theory was that some function of the spleen had depressed haematopoiesis in the marrow, and it was felt that compensatory haematopoiesis in the spleen was relatively unimportant. The offending organ, namely the spleen, was therefore removed, and six weeks later the patient was doing well and the blood count was improving.

*Question:* What is known about the mechanism of the bone marrow for holding on to immature cells?

*Dr. F. J. W. Lewis:* One view that has been expressed is that very immature cells tend to stick together and not move into the peripheral blood.

Dr. Lloyd then asked Mr. Cooke if he had any experience of removing spleens in these cases.

*Mr. Cooke:* Splenectomy can be a hazardous operation and I would have to be convinced that it would be really helpful, or that the spleen was doing secondary harm before you would make me do it. I remember some time ago a very capable surgeon showing a case like this the day before he was going to operate and I remember saying to myself, " This is an oldish lady and if she comes to any harm I shall feel something wrong has been done." We saw the operation and by the time I left the theatre there was blood everywhere, three limbs were being used for intra-arterial transfusion and there was a " dead body " on the table. I think if you are talking about splenectomy you have to think twice before operating: I have no personal experience of doing it for this particular condition.

PLATE I

*Sternum, inner aspect of cortex. The arrows show a "reversal line". This is a cement line which marks the inner limit of the normal cortex. The arborizing osteophytes attached to it are due to myelosclerosis, and the fibrous tissue out of which they are formed can be seen to the right of centre* (× 110).

*Sternum, medullary trabeculae. These are coarse and thick* (× 110).

*Prostate.* *A well-differentiated adenocarcinoma with small acini is seen invading the parallel fibres of collagen and smooth muscle which form the capsule of the gland* (× 200).

## Figures and Tables

**Figure f1:**
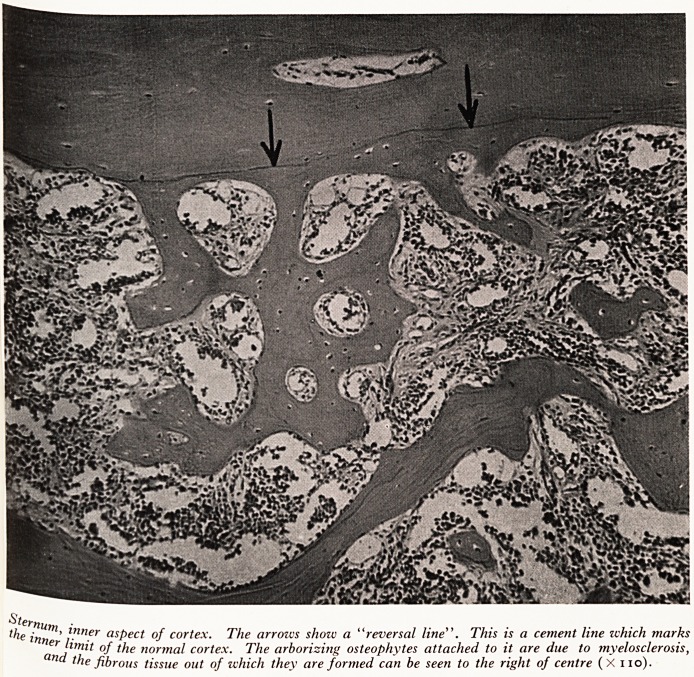


**Figure f2:**
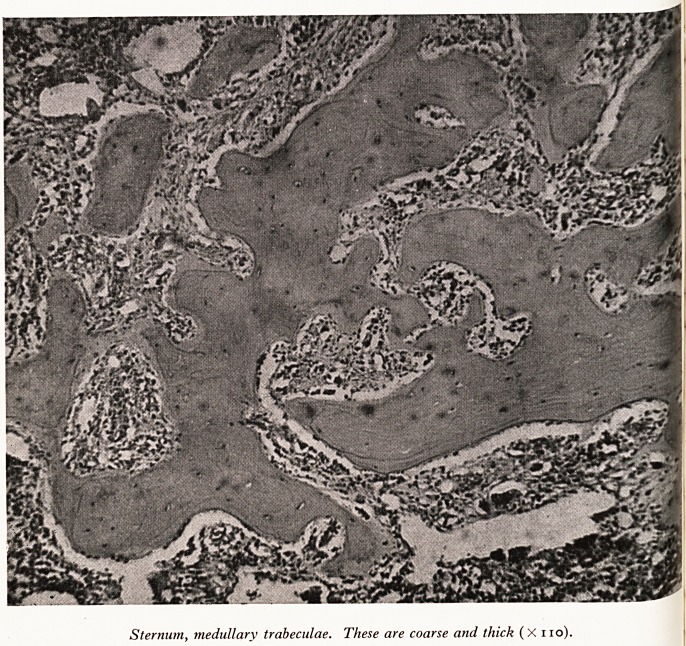


**Figure f3:**